# Individual dispersal decisions affect fitness via maternal rank effects in male rhesus macaques

**DOI:** 10.1038/srep32212

**Published:** 2016-08-31

**Authors:** Brigitte M. Weiß, Lars Kulik, Angelina V. Ruiz-Lambides, Anja Widdig

**Affiliations:** 1 Behavioural Ecology Research Group, Institute of Biology, University of Leipzig, Germany; 2 Junior Research Group of Primate Kin Selection, Department of Primatology, Max-Planck-Institute for Evolutionary Anthropology, Germany; 3 Caribbean Primate Research Center Cayo Santiago, University of Puerto Rico, Punta Santiago, USA

## Abstract

Natal dispersal may have considerable social, ecological and evolutionary consequences. While species-specific dispersal strategies have received much attention, individual variation in dispersal decisions and its fitness consequences remain poorly understood. We investigated causes and consequences of natal dispersal age in rhesus macaques (*Macaca mulatta*), a species with male dispersal. Using long-term demographic and genetic data from a semi-free ranging population on Cayo Santiago, Puerto Rico, we analysed how the social environment such as maternal family, group and population characteristics affected the age at which males leave their natal group. While natal dispersal age was unrelated to most measures of group or population structure, our study confirmed earlier findings that sons of high-ranking mothers dispersed later than sons of low-ranking ones. Natal dispersal age did not affect males’ subsequent survival, but males dispersing later were more likely to reproduce. Late dispersers were likely to start reproducing while still residing in their natal group, frequently produced extra-group offspring before natal dispersal and subsequently dispersed to the group in which they had fathered offspring more likely than expected. Hence, the timing of natal dispersal was affected by maternal rank and influenced male reproduction, which, in turn affected which group males dispersed to.

Natal dispersal has a major impact on the social dynamics and genetic structure of populations^1^ and factors influencing individual dispersal decisions may have considerable social, ecological and evolutionary consequences^2^. Accordingly, dispersal has received broad interest in evolutionary ecology^3^, but it is inherently difficult to quantify due to the challenges posed by following individuals after dispersal^4^,^5^. Hence, while species-typical patterns such as dispersal rates or sex-bias in dispersal are well-studied, individual-based approaches to animal dispersal are relatively scarce and there remains uncertainty about how individual traits mediate dispersal and its effects^3^,^4^.

Yet, a growing number of studies show within-population variation in natal dispersal^6^, shifting attention from considering dispersal as an inflexible process to one that varies between individuals^7^. Recent research in birds and mammals presents a complex picture where individuals adjust their natal dispersal strategies according to individual, social and ecological conditions^2^,^8^,^9^,^10^. In rodents, for instance, the likelihood of dispersing from the natal range was found to be influenced by factors such as resource availability^11^, group size^12^ or interactions among close relatives^2^. Avian dispersal has been related to clutch size, hatching date, resource availability and fledgling mass^13^ but also to animal personality^9^,^13^ or the degree to which an individual was inbred^3^. In primates, leaving or staying in the natal group has been related to group size and composition, whereby male dispersal in many species seems to be driven by the resulting mating opportunities (e.g. white-faced capuchins *Cebus capucinus*^14^; savanna baboons *Papio cynocephalus spp*^15^; black-faced lion tamarins *Leontopithecus caissara*^16^). In female-dispersing mountain gorillas (*Gorilla beringei beringei*), on the other hand, natal dispersal appeared to be driven by infanticide avoidance, with natal females being more likely to leave one-male groups, where infanticide risk is higher than in multi-male groups^17^.

The individual fitness consequences of dispersal decisions may vary depending on the costs and benefits of dispersal imposed by an individual’s state and environment^5^,^7^. Quantifying these consequences poses a particular challenge because of the difficulty in monitoring how individuals fare after dispersal. Evidence for a reduction in fitness imposed by dispersal comes from Mauritius kestrels (*Falco punctatus*), where females, but not males, dispersing farther from their natal territory showed reduced fecundity early in life, more rapid aging later in life and, consequently, lower lifetime reproductive success^18^. Other studies in birds point towards increased reproductive opportunities and lifetime reproductive success associated with delayed natal dispersal (Siberian jays *Perisoreus infaustus*^19^; Seychelles warblers *Acrocephalus sechellensis*^20^). Siberian jays dispersing later also experienced enhanced survival in a study by Griesser *et al*.^21^, although an effect of delayed dispersal on survival could not be observed in an earlier study on the same species^19^. Evidence for an effect of dispersal on survival has also been mixed in rodents, with some studies describing reduced survival of dispersers compared to philopatric individuals and others finding no relationship between dispersal and survival, but none describing increased survival of dispersers (reviewed in ref. 22). In banner-tailed kangaroo rats (*Dipodomys spectabilis*), dispersal also had no measurable effects on fecundity or the probability of recruiting offspring^23^ (but see ref. 24), while in spotted hyenas (*Crocuta crocuta*) the choice where to disperse to affected male reproductive success and the authors suggest that in this female-dominated species female mate choice and the resulting reproductive opportunities drive male dispersal^8^.

The present study investigated the social mediators and lifetime fitness consequences of natal dispersal decisions in the rhesus macaques of Cayo Santiago, a population that is ideally suited for studying dispersal. The animals are roaming freely on a 15 ha island, which allows reliably tracking all individuals throughout their lives. Dispersal data along with detailed life histories and genetically determined parentage are available for a large number of individuals.

Rhesus macaques live in multi-male, multi-female groups, with most males dispersing from their natal group around puberty, i.e. when they are four to five years old^25^,^26^. Dispersal in this species is seasonal, predominantly occurring during the mating season^25^,^27^, and males may continue to change groups throughout their lives^25^. Natal dispersal age may vary considerably, with some individuals dispersing as early as 2.5 years of age and others remaining in their natal group beyond an age of eight years^26^. On Cayo Santiago, males have an average life span of 12 years but can get more than 20 years old^28^, whereby natal dispersal was associated with a 6% mortality rate^29^. Early studies on a small number of rhesus macaques ranging freely at La Parguera, Puerto Rico, indicated that males frequently dispersed with peers and tended to disperse into groups with a higher rate of females^25^ or into groups containing older maternal brothers^30^. Natal dispersal age at La Parguera appeared to be unaffected by population size, whether or not males were orphaned and whether mothers were high, medium or low-ranking^25^. A later study, conducted on Cayo Santiago on a larger sample of males, described that males from high-ranking lineages dispersed later than those from low-ranking ones^26^. This study further indicated that a weak mother-son relationship may be predictive of early dispersal^26^, a finding that was recently substantiated in a study on the development of mother-offspring bonds in rhesus macaques^31^. The regulation of natal dispersal appears to involve serotonin, as indicated by the relationship between natal dispersal age and serotonin transporter genotype^32^. Interestingly, the serotonin pathway is also involved in male reproductive timing, indicating that male reproduction and natal dispersal age might be intrinsically linked^33^.

To better understand the social mechanisms mediating natal dispersal and its fitness effects we explored individual variation in natal dispersal age in a powerful long-term data set of 45 years on over 900 individual males. Building upon earlier studies, we assessed the effects of the population, group and maternal environment on natal dispersal age within one analytical framework. We further related natal dispersal age and its drivers to short-term and long-term consequences on reproduction and survival, allowing us to investigate the fitness consequences of natal dispersal decisions and to disentangle whether these were mediated directly by natal dispersal age or by shared social drivers. Finally, we investigated how reproductive patterns mediated by the timing of natal dispersal affected the decision where to disperse to.

## Results

### Variation in natal dispersal age

**Figure 1:**
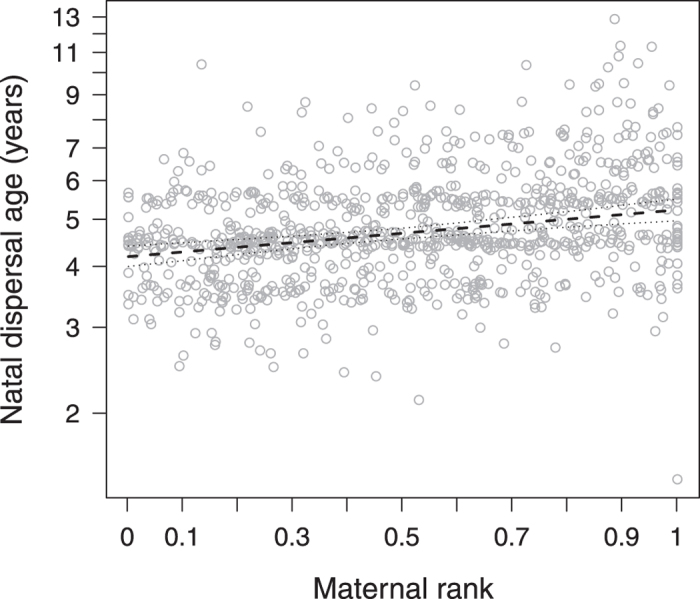
Natal dispersal age (years) as a function of maternal rank (from 0 = low to 1 = high). Points depict the raw data, lines show the fitted model (dashed) and 95% confidence limits (dotted) conditional on the other predictors being at their average.

**Figure 2:**
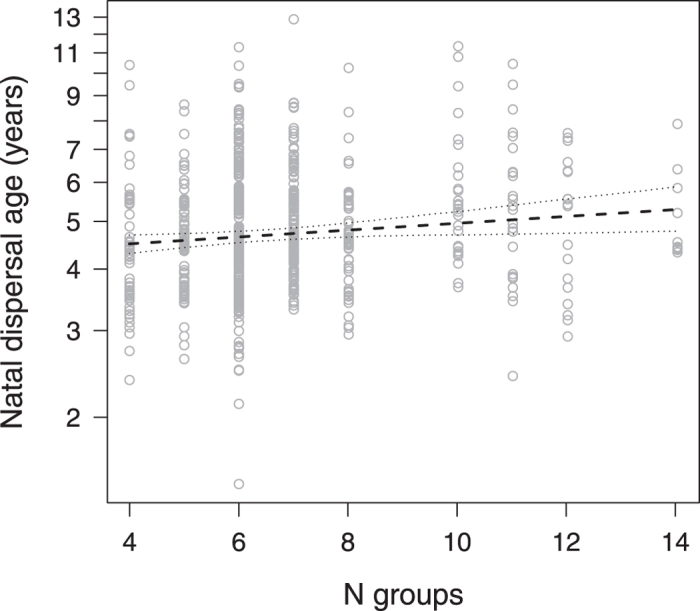
Natal dispersal age (years) as a function of the number of groups available for dispersal. Points depict the raw data, lines show the fitted model (dashed) and 95% confidence limits (dotted) conditional on the other predictors being at their average.

The 912 focal males dispersed from their natal group at a mean age of 4.9 ± 1.3 (mean ± SD) years. The social environment significantly contributed to the observed variation in natal dispersal age (full-null model comparison, LRT: χ^2^ = 25.541, df = 9, P = 0.002). In particular, sons of high-ranking mothers dispersed, on average, one year later than sons of low-ranking mothers (Table 1, Fig. 1). Sons who lost their mother before maturation tended to disperse earlier than those whose mother was present throughout both infancy and early puberty (Table 1). Furthermore, males dispersed from their natal group at a later age if more groups were present on Cayo Santiago (Table 1, Fig. 2). Neither population size, natal group sex ratio or group size, the number of older siblings, the presence of familiar older brothers, maternal family size nor any of the interactions significantly explained variation in natal dispersal age in our data set (Table 1).

### Natal dispersal age and fitness

Natal dispersal age did not affect the likelihood to survive the first year after dispersal (GLMM, N = 840, estimate ± SE = 0.00008 ± 0.0006, z = 0.12, P = 0.898), nor was it related to an individual’s post-dispersal longevity (GLMM, N = 402, estimate ± SE = −0.0001 ± 0.0003, z = −0.47, P = 0.639).

**Figure 3:**
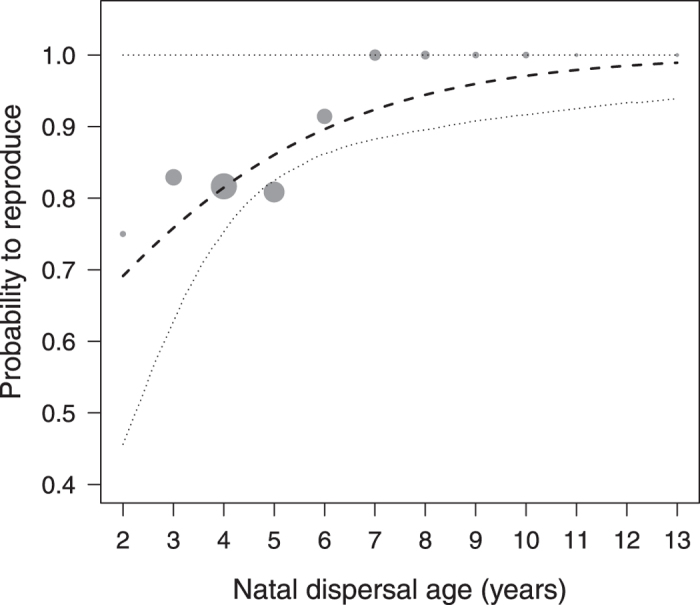
Probability to reproduce over life as a function of natal dispersal age (years). Points show proportions of males reproducing with natal dispersal age binned into ~ yearly sections. The area of the points corresponds to the respective sample size (1 to 109 males per bin), lines show the fitted model (dashed) and 95% confidence limits (dotted) conditional on the control predictors being at their average.

**Figure 4:**
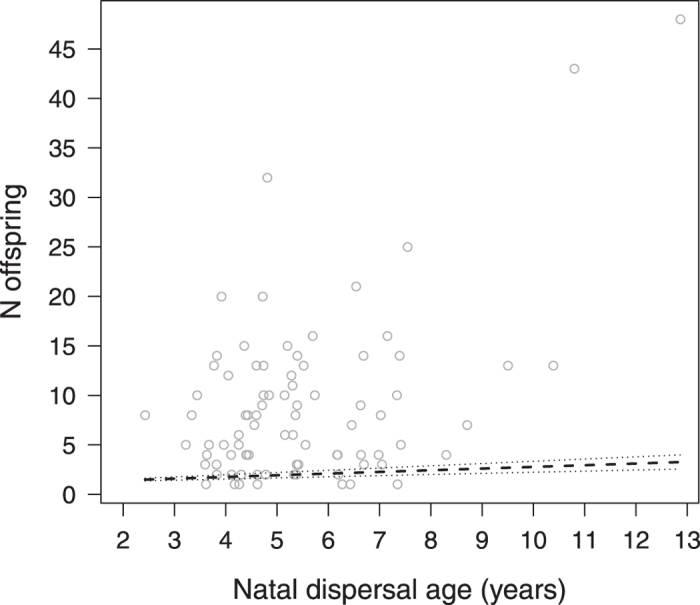
Lifetime reproductive success (number of offspring) as a function of natal dispersal age (years). Points depict the raw data, lines show the fitted model (dashed) and 95% confidence limits (dotted) conditional on the control predictors being at their average.

Males who dispersed from their natal group at a later age had a higher likelihood of siring offspring over their lifetime than those dispersing at a younger age, whereby variation was large among early dispersers, while all of the males with complete life histories that dispersed beyond the age of seven years sired at least one offspring in their lifetime (GLMM, N = 297, estimate ± SE = 0.001 ± 0.0004, z = 2.42, P = 0.015, Fig. 3). Natal dispersal age was not significantly related to the onset of reproduction (GLMM, N = 252, estimate ± SE = −0.00003 ± 0.00004, z = −0.75, P = 0.454). Among those males who did reproduce, late dispersers produced more offspring over their lifetime than early dispersers (GLMM, N = 84, estimate ± SE = 0.0005 ± 0.0001, z = 3.65, P < 0.001, Fig. 4). LRS was additionally affected by maternal rank (GLMM, N = 84, estimate ± SE = 0.7064 ± 0.2917, z = 2.42, P = 0.016), while neither maternal rank nor co-residence affected any of the other fitness parameters (for summary of results see Supplementary Table S1).

### Natal dispersal age, place of reproduction and group choice

**Figure 5:**
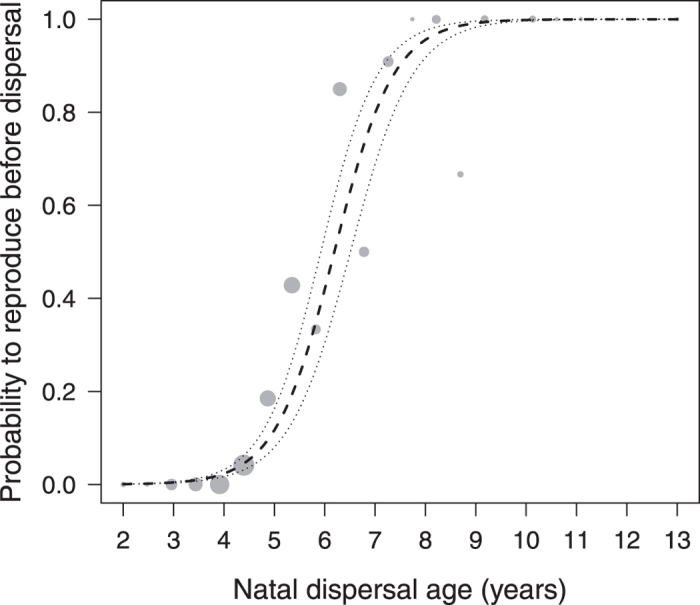
Probability to start reproduction before natal dispersal as a function of natal dispersal age (years). Data comprise only males that did reproduce, with points showing proportions of males starting to reproduce before natal dispersal per natal dispersal age bin. The area of the points corresponds to the respective sample size (1 to 48 males per bin), lines show the fitted model (dashed) and 95% confidence limits (dotted).

**Figure 6:**
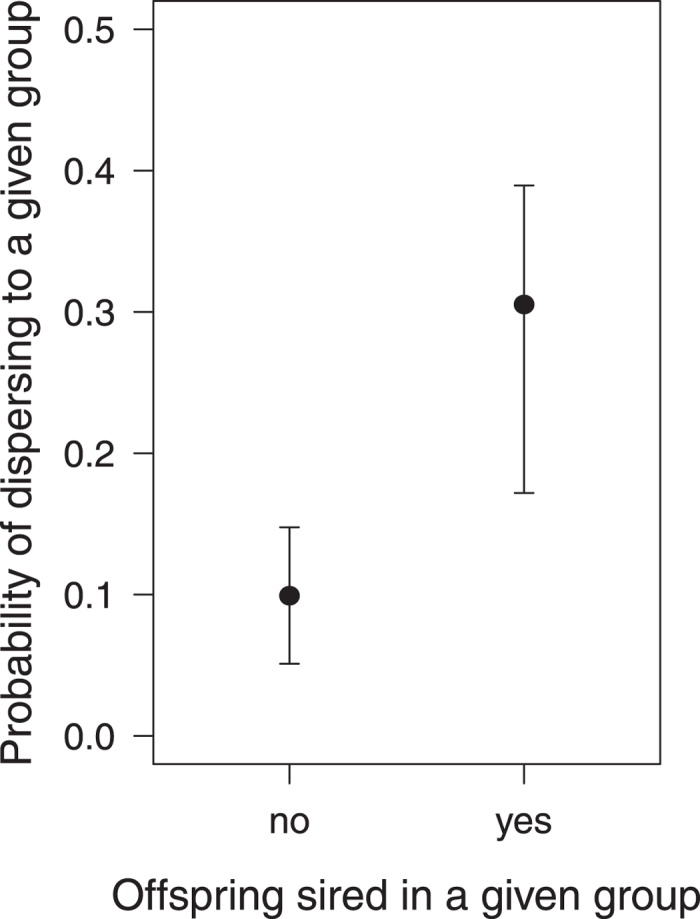
Probability to disperse to a certain group as a function of whether a male sired offspring in the respective group or not. Circles show the fitted model, whiskers 95% confidence intervals.

Of 252 males that did reproduce over their lifetime, 76 (30.2%) started to do so while still residing in their natal group, whereby late dispersers were more likely to start reproducing in their natal group than early dispersers (GLMM, N = 253, estimate ± SE = 0.005 ± 0.0006, z = 7.36, P < 0.001, Fig. 5). Notably, 69% of the males reproducing before dispersal sired offspring in other social groups (extra-group paternity). These extra-group sires subsequently were more likely to disperse to a group in which they had fathered offspring than to a group in which they had not done so (GLMM, N = 400 potential groups for 65 males, estimate ± SE = 1.12 ± 0.45, z = 2.49, P = 0.013, Fig. 6).

## Discussion

Based on 45 years of demographic and 22 years of genetic data on multiple social groups of rhesus macaques, the present study provides the first comprehensive analysis of the socioecological causes in conjunction with the fitness consequences of individual dispersal decisions in a non-human primate. Natal dispersal age was clearly influenced by maternal rank, confirming earlier findings in the same population using a smaller sample^26^. Furthermore, natal dispersal age appeared to be affected by the number of social groups in the population and by the mother’s presence up to adulthood, while none of the other characteristics we tested influenced the timing of departure. We detected no measurable consequences of natal dispersal age on individual survival, but both the likelihood to reproduce as well as lifetime reproductive success were affected by the age at which males left their natal group. Notably, the timing of natal dispersal influenced where males reproduced and where they subsequently dispersed to.

The social environment has been identified as a factor that may affect dispersal decisions in a range of species. In the present study, the most notable social driver of natal dispersal was the dominance rank of a male’s mother, with high-born sons dispersing from their natal group on average one year later than low-born sons (see Fig. 1). Our longitudinal study thus confirms an earlier study on ~200 individuals from the Cayo Santiago population describing that males from high-ranking lineages dispersed later than those from low-ranking ones^26^. Males in our study also tended to disperse sooner if they lost their mother within the first three years of life. Several studies in birds and mammals previously suggested that parental presence on the natal territory may delay offspring dispersal (e.g. Seychelles warblers^20^, Japanese field mice *Apodemus speciosus*^2^, savanna baboons^34^ but see refs 15,25). Furthermore, the natal territory has been suggested to serve as a safe haven for young offspring^19^ until reproductive opportunities arise. Particularly if rhesus mothers are high-ranking, they could constitute this “haven” and provide their sons with the silver spoon effect^35^ associated with a privileged upbringing. In rhesus macaques, maternal rank is highly predictive of offspring rank in the natal group^36^, and high-ranking males indeed were described to sexually matured faster than low-ranking ones^37^. Interestingly, also in male-philopatric bonobos (*Pan paniscus*), sons of high-ranking females attained the highest paternity success^38^ and the presence of mothers enhanced their sons’ mating success^39^. Other than the rank and presence of the mother, however, the availability of kin appeared to have no influence on natal dispersal age in our study.

Variation in natal dispersal has frequently been related to group characteristics and population density (e.g. degus *Octodon degus*^12^; African lions *Panthera leo*^40^; feral horses *Equus ferus caballus*^10^), although the direction of the relationship (positive or negative) may vary considerably depending on the social structure and environmental conditions encountered^41^,^42^. In the present study neither population size, group size, nor sex ratio in the natal group affected natal dispersal age, although males appeared to leave their natal group slightly later if a larger number of groups was available for dispersal. Altogether, these results rather seem to contradict the notion that dispersal is a function of limited reproductive opportunities. However, parameters like group size and sex ratio may not only depend on the conditions in the natal group but on how they compare to the respective conditions in non-natal groups. Furthermore, reproductive opportunities could arise from more than sheer numbers through e.g., partner preferences or familiarity, as female mate choice has been shown to contribute to male reproductive success in macaques^43^.

In the present study, natal dispersal age was not related to short-term or long-term survival. However, only 9% of the males in the present study died within the first year after natal dispersal, similar to the dispersal-related mortality rate of 6% reported in an earlier study on the same population^29^. This low mortality could be related to the high familiarity of males with their habitat as well as supplemental feeding on Cayo Santiago, although males are still likely to face the social costs of dispersal due to loosing social ties and rank^44^, including reduced access to the supplemental food sources. These costs, however, either do not differ between early or late dispersers or are too insubstantial to affect survival in the study population. Notably, also studies in some bird species did not detect a relationship between survival and variation in natal dispersal^18^,^19^ (but see ref. 21).

Several studies, predominantly in birds, have provided evidence that delayed dispersal may enhance reproductive success^19^,^20^. Our results indicate that also late-dispersing rhesus males were more likely to sire offspring in their lifetime and overall had a slightly higher lifetime reproductive success than early dispersers. Some of the late dispersers, however, had exceptionally high reproductive success, siring over 40 offspring, and produced the majority of their offspring while still residing in their natal group. Although dispersing animals typically leave their natal group or territory to breed elsewhere, a subset of individuals may initiate their reproductive career before natal dispersal (e.g. spotted hyenas^8^; primates^15^,^42^). In the Cayo Santiago rhesus macaques, 30% of the males sired offspring while still residing in their natal group, and these were particularly the late dispersers. It is unlikely that successful reproduction in the natal group primarily made males delay dispersal, as the average onset of male reproduction (7.2 ± 1.9 years) was well after the average age of natal dispersal (4.9 ± 1.3 years). Rather, early and late dispersers may pursue different reproductive tactics conditional on maternal rank^45^. Rhesus males attain a rank similar to their mother in their natal group, but start at the bottom of the hierarchy when changing to a new group, where they increase in rank by queuing^46^,^47^. Accordingly, staying longer in the natal group may allow sons of high-ranking mothers to initiate their reproductive career in a social setting in which they can benefit from their well-established social bonds and high rank, while low-born sons may be better off if they disperse early and queue for reproductive opportunities elsewhere. As such, our results are in line with earlier studies in this population that suggested that high-ranking and low-ranking males pursue different reproductive strategies^45^, with the strategies of high-ranking males sometimes, but not always, resulting in higher reproductive success (see overview in ref. 48). Notably, also in other macaque species, rank-dependent reproductive strategies were related to reproductive success in some, but not in other studies^49^,^50^ (see overview in ref. 51), which has been suggested to be partly due to the role of female choice in macaque mating systems^43^. In the present study, maternal rank did affect male LRS but not the probability to reproduce *per se*; yet natal dispersal age affected both reproductive traits even though maternal rank and co-residence between mothers and sons were statistically controlled for. Hence, maternal rank contributed to reproductive success but was not the sole cause for the relationship between natal dispersal age and successful reproduction.

Finally, pursuing a strategy of breeding in the natal group carries an increased risk of inbreeding, which may be alleviated by seeking reproductive partners outside the group, i.e. extra-group mating^52^. Extra-group paternity (EGP) is common in mammals, accounting for a mean of 29.2% of offspring in 20 of 26 investigated species^53^; in rhesus macaques, levels of EGP range from 16–36% (Ruiz-Lambides unpublished data)^53^,^54^. In the present study, more than two thirds of the males reproducing before natal dispersal engaged in EGPs and these males were more likely to disperse to a group in which they had fathered an infant than to one in which they had not. One motivation for such a dispersal decision could be the incentive to provide paternal care, as rhesus males were shown to affiliate more with their offspring than with unrelated infants^55^. In addition, also a female preference for novel males has been suggested to be partly associated with male group transfer (see ref. 48), however, males who already sired offspring in a group before they dispersed into it are probably already somewhat familiar with its group members. Yet, dispersing to a group with some familiar females could be beneficial for both sexes, as non-sexual male-female bonds have been suggested to be more important for female fitness than the choice of mating partners^48^. For dispersing males, familiar group members (e.g. the mothers of their offspring) could alleviate the social costs associated with group transfer, as recently demonstrated in cooperatively breeding cichlids^56^.

In conclusion, our longitudinal study confirmed earlier findings^26^ that sons of high-ranking mothers dispersed from their natal group at older ages than those of low-ranking mothers and is also in line with previous studies^45^,^46^ linking different ranks with different reproductive tactics. Males that delayed dispersal benefitted in terms of more successful reproduction than early dispersers, which appeared to be achieved by reproducing in their natal group or as extra-group males prior to dispersal. Siring extra-group offspring, in turn, interacted with the decision where to disperse to, which is likely to have profound effects on an individual’s future social life. Hence, maternal rank directly and – via affecting natal dispersal age – indirectly affected reproductive success and reproductive patterns in rhesus macaques and thus is at the core of the complex interplay between natal dispersal and reproduction.

## Methods

### Study population

Data were collected on a free-ranging population of rhesus macaques at Cayo Santiago (CS, 18°09′N, 65°44′W), Puerto Rico. The population was established in 1938 by introducing 409 individuals caught in various locations in India^57^. No other individuals have been added to the population since except through natural births, and genetic analyses show that inbreeding is no issue (A. Widdig, unpublished data). Over the time period considered in this study (1969 to 2014), population size has averaged ~800 ± 260 (mean ± SD, range 189–1299) individuals living in 7 ± 2 social groups (mean ± SD, range 4–14).

The population is managed by the Caribbean Primate Research Center (CPRC) and is partly provisioned with commercial high-protein biscuits every morning (0.23 kg/monkey/day). Foraging on natural vegetation and insects accounts for ~50% of the foraging time^58^. Water is provided *ad libitum* in drinking basins. Individuals are tattooed for identification purposes and receive a tetanus primary immunization and booster as yearlings and two-year-olds, respectively. There is no other medical intervention on the population. Population size is controlled via a mixture of natural death and culling strategies, including the removal of entire groups or randomly culling individuals younger than three years old (see ref. 59 for details).

### Long-term demographic data and study animals

The CPRC maintains a detailed demographic database based on records of daily censuses conducted continuously since 1956. The database contains individual information on births and deaths, sex, maternal relatedness and group membership. Data on all live births, deaths, and group changes are typically reported immediately or within 2 days from occurrence. Male dispersal is only assigned once residency remains constant for at least 30 days. Once stable, the first day a male was seen in the new group is assigned as the date of immigration.

For this study we used demographic data of 921 males from the cohorts 1969 to 2009 whose mothers were known and who dispersed from their natal groups between 1972 and 2014. We did not use data from earlier cohorts to avoid inconsistencies arising from different census formats and none of later cohorts to avoid a bias towards particularly young dispersers. We further excluded males who dispersed with their mothers in the process of group fissions before the age of five years or who dispersed while their birth group was being removed from the island.

### Dominance rank data

Systematic data on male dominance rank spanning the entire study period and population are not available. As in other cercopithecines, however, rhesus offspring rank directly below their mothers in reverse birth order^36^,^60^ and we thus used maternal rank as a proxy (see ref. 61) of male rank before natal dispersal. Individual maternal ranks were obtained by using long-term observations of matrilineal ranks from 1970 to present, confirmed with detailed observations of dyadic interactions where available. As dominance relationships among adult females are highly stable over time^62^, we used these data to calculate daily individual maternal ranks, which were updated with any changes in dominance rank brought about by births and deaths using a self-written R script. We standardized maternal ranks per day to a range from 0 to 1 (lowest to highest ranking).

### Parentage assignment

A genetic database of the population was implemented in 1992 and DNA (from blood, tissue or faeces) of nearly the entire population has been sampled since. At present, genetic information is available for 4641 animals, genotyped for an average of 27.6 ± 1.6 microsatellite markers (details in ref. 63, Widdig *et al*. in revision). Paternity could be determined for 3934 out of 4014 individuals sampled between 1992 and 2014 (98.0%) by using a combination of exclusion and likelihood analyses, considering as potential sires all mature males present on the island around conception of a given infant^28^ (see ref. 63). Maternity was derived from census records and could be confirmed genetically for 3946 (98.7%) of the 4000 mother-offspring pairs sampled (Widdig *et al*. in revision). In the present data set, maternity was determined genetically for 394 of the focal males, whereby the census mother could be confirmed in 387 (98.8%) of the cases. In the remaining seven cases, we assigned the genetically determined mother as the focal’s mother.

### Statistical analysis

To assess the effects of predictor variables conditional on potential effects of other predictors and to avoid multiple testing issues we fitted all predictor variables for a given response into a Linear Mixed Model (LMM) or Generalized Linear Mixed Model (GLMM) in R 3.0.2^64^. To identify variables related to variation in dispersal age we calculated an LMM with log-transformed natal dispersal age (in days) as the Gaussian response variable. We fitted test predictors related to the focal males’ population, group and maternal environment, i.e. population size, the number of groups on Cayo Santiago, adult group size of the natal group, adult sex ratio of the natal group, maternal rank and maternal family size (see Table 1, Supplementary methods). We further scored how many days the mother was alive and present in the first 1000 days of a male’s life and, as a proxy for maternal experience, the number of offspring produced by the mother until the male’s birth. As we expected that familiar male kin in other groups could be an incentive for dispersal, we further scored if the focal had familiar older brothers that had already dispersed when it reached dispersal age (i.e. three years of age). We also fitted interactions between group size and sex ratio, group size and maternal family size and between population size and the number of groups on Cayo Santiago. To control for the possibility that males born early in the season would disperse earlier than late-born ones we fitted the age at the onset of the mating season in the focal’s third year of life as a fixed effects control predictor. We further fitted the identity of the mother, the male’s birth group and cohort as random effects.

All covariates were z-transformed to a mean of zero and a standard deviation of one to get comparable estimates and facilitate model convergence. We removed interactions with a p-value < 0.1 from the model to facilitate the interpretation of main terms but kept all other terms in the model.

We investigated the effects of natal dispersal age on five fitness traits: (i) if the male survived the first year after natal dispersal, (ii) post-dispersal longevity (i.e. how long the male survived after dispersal), (iii) the probability to reproduce (i.e. if a male reproduced in his lifetime), (iv) age at first reproduction and (v) lifetime reproductive success (LRS, i.e. number of offspring sired over lifetime). We calculated GLMMs with surviving the first year after dispersal and the probability to reproduce as binomial response variables; post-dispersal longevity (in years), age at first reproduction (in years) and LRS were fitted with a Poisson error structure. Natal dispersal age (in days) was fitted as the only test predictor. As control predictors we included individual traits that tended to affect natal dispersal age in order to assess whether fitness traits were affected by natal dispersal age or a phenotypically correlated trait (see Supplementary Table S1) and fitted identity of the mother, the male’s birth group and cohort as random effects.

To investigate the relationship between natal dispersal age and where males first reproduced we fitted a GLMM with pre- or post-dispersal onset of reproduction as the binomial response variable and natal dispersal age as the only test predictor. As controls we fitted the identity of the male’s mother, birth group and cohort as a random effect. For males starting to reproduce before natal dispersal we determined if they had sired offspring with females from their natal group (i.e. within-group offspring) or females residing in another group than the focal male on the day the infant was conceived (i.e. extra-group offspring). To test if having fathered extra-group offspring affected group choice during natal dispersal we determined all groups available to a male on the day of his natal dispersal and scored if the male immigrated into a given group or not. We fitted a GLMM with group choice as the binomial response variable and having sired offspring in the respective group (yes/no) prior to dispersal as the only test predictor. We controlled for repeated measures per individual by fitting male ID as a random effect.

For all models we fitted random slopes for all test predictors that varied within each level of a random effect to achieve reliable p-values^65^ (see Table 1 and Supplementary Table S1). All models fulfilled the assumptions of the respective distributions and showed no evidence of collinearity or model instability (see Supplementary methods). In all cases we assessed the significance of the full model by comparing it to a null model comprising only control predictors, random intercepts and random slopes using a Likelihood Ratio Test.

### Ethics statement

All research procedures were approved by the CPRC and the Institutional Animal Care and Use Committee (IACUC) of the University of Puerto Rico (protocol number 4060105). The transfer of samples for paternity analyses was approved by Cites Export permission #05US101361/9, #06US112079/9, #07US133766/9, #08US163309/9, #09US200870/9, #09US230435/9, #11US28371A/9 and Cites Import permission #E-1426/05, #E-1082/06, #E-1207/07, #E-1215/08, #E01146/09, #E-00049/10, #E-00836/11). All methods were performed in accordance with the relevant guidelines and regulations.

## Additional Information

**How to cite this article**: Weiß, B. M. *et al*. Individual dispersal decisions affect fitness via maternal rank effects in male rhesus macaques. *Sci. Rep.* **6**, 32212; doi: 10.1038/srep32212 (2016).

## Supplementary Material

Supplementary Information

## Figures and Tables

**Table 1:** Results of the LMM with dispersal age (in days) as the Gaussian response variable.

	Predictor	Estimate	SE	χ^2^	P
	Intercept	7.4483	0.013		
population environment	population size	0.0239	0.015	2.009	0.156
	**n groups**	**0.0283**	**0.011**	**4.147**	**0.042**
group environment	sex ratio	0.0057	0.015	0.106	0.745
	group size	−0.0172	0.014	1.387	0.239
maternal/family environment	**maternal rank**	**0.0643**	**0.013**	**11.943**	**<0.001**
	n^th^ offspring	0.0097	0.0097	0.951	0.329
	dispersed brother (yes)	−0.0192	0.021	0.770	0.380
	maternal family size	−0.0082	0.012	0.449	0.503
	***co-residence with mother***	***0.0148***	***0.008***	***3.477***	***0.062***
control	age at onset of 3^rd^ mating season	−0.0028	0.008	0.107	0.743

“Co-residence with mother” was measured as the number of days in a male’s first 1000 days of life that his mother was alive. “Age at onset of 3^rd^ mating season” refers to a male’s age at the onset of the mating season in his third year of life and is a proxy for whether the male was born early or late in the season (see methods for details). SE = standard error.

Random intercepts were fitted for maternal ID, birth group and cohort, random slopes for sex ratio, group size, maternal rank, n^th^ offspring, maternal family size and age 3^rd^ season within birth group and cohort and for population size and n groups within birth group. Interactions group size*sex ratio, group size*family size, population size*n groups had p-values > 0.1 and were removed from the model.

## References

[b1] Johnson M. L. & Gaines M. S. Evolution of Dispersal: Theoretical Models and Empirical Tests Using Birds and Mammals. Annu. Rev. Ecol. Syst. 21, 449–480 (1990).

[b2] Sakamoto S. . The effects of maternal presence on natal dispersal are seasonally flexible in an asocial rodent. Behav. Ecol. Sociobiol. 69, 1075–1084 (2015).

[b3] Hardouin L. A., Legagneux P., Hingrat Y. & Robert A. Sex-specific dispersal responses to inbreeding and kinship. Anim. Behav. 105, 1–10 (2015).

[b4] Lowe W. H. & McPeek M. A. Is dispersal neutral? Trends Ecol. Evol. 29, 444–450 (2014). 24962790 10.1016/j.tree.2014.05.009

[b5] Doligez B. & Pärt T. Estimating fitness consequences of dispersal: a road to ‘know-where’? Non-random dispersal and the underestimation of dispersers’ fitness. J. Anim. Ecol. 77, 1199–1211 (2008). 18808435 10.1111/j.1365-2656.2008.01446.x

[b6] Wikberg E. C., Sicotte P., Campos F. A. & Ting N. Between-Group Variation in Female Dispersal, Kin Composition of Groups, and Proximity Patterns in a Black-and-White Colobus Monkey (Colobus vellerosus). PLoS ONE 7, e48740 (2012). 23144951 10.1371/journal.pone.0048740 PMC3492432

[b7] Tarwater C. E. & Beissinger S. R. Dispersal polymorphisms from natal phenotype–environment interactions have carry-over effects on lifetime reproductive success of a tropical parrot. Ecol. Lett. 15, 1218–1229 (2012). 22906164 10.1111/j.1461-0248.2012.01843.x

[b8] Höner O. P. . Female mate-choice drives the evolution of male-biased dispersal in a social mammal. Nature 448, 798–801 (2007). 17700698 10.1038/nature06040

[b9] Aguillon S. & Duckworth R. Kin aggression and resource availability influence phenotype-dependent dispersal in a passerine bird. Behav. Ecol. Sociobiol. 69, 625–633 (2015).

[b10] Marjamäki P. H., Contasti A. L., Coulson T. N. & McLoughlin P. D. Local density and group size interacts with age and sex to determine direction and rate of social dispersal in a polygynous mammal. Ecol. Evol. 3, 3073–3082 (2013). 24101995 10.1002/ece3.694 PMC3790552

[b11] Edelman A. J. Sex-specific effects of size and condition on timing of natal dispersal in kangaroo rats. Behav. Ecol. 22, 776–783 (2011).

[b12] Quirici V., Faugeron S., Hayes L. D. & Ebensperger L. A. The influence of group size on natal dispersal in the communally rearing and semifossorial rodent, Octodon degus. Behav. Ecol. Sociobiol. 65, 787–798 (2011).

[b13] Dingemanse N. J., Both C., van Noordwijk A. J., Rutten A. L. & Drent P. J. Natal dispersal and personalities in great tits (Parus major) 270 (2003). 10.1098/rspb.2002.2300 PMC1691302 12713749

[b14] Jack K. M., Sheller C. & Fedigan L. M. Social factors influencing natal dispersal in male white-faced capuchins (Cebus capucinus). Am. J. Primatol. 74, 359–365 (2012). 21732399 10.1002/ajp.20974

[b15] Onyango P. O., Gesquiere L. R., Altmann J. & Alberts S. C. Puberty and dispersal in a wild primate population. Horm. Behav. 64, 240–249 (2013). 23998668 10.1016/j.yhbeh.2013.02.014 PMC3764504

[b16] Nascimento A. T. A., Nali C. & da Fonseca G. A. B. Dispersal, Group Formation and Kinship in the Black-Faced Lion Tamarin (Leontopithecus caissara). Folia Primatol. (Basel) 85, 216–227 (2014). 25301031 10.1159/000363058

[b17] Robbins A., Stoinski T., Fawcett K. & Robbins M. Socioecological influences on the dispersal of female mountain gorillas—evidence of a second folivore paradox. Behav. Ecol. Sociobiol. 63, 477–489 (2009).

[b18] Nevoux M., Arlt D., Nicoll M., Jones C. & Norris K. The short- and long-term fitness consequences of natal dispersal in a wild bird population. Ecol. Lett. 16, 438–445 (2013). 23360587 10.1111/ele.12060

[b19] Ekman J., Bylin A. & Tegelström H. Increased lifetime reproductive success for Siberian jay (Perisoreus infaustus) males with delayed dispersal. Proc. R. Soc. B 266, 911–915 (1999).

[b20] Eikenaar C., Richardson D., Brouwer L. & Komdeur J. Parent presence, delayed dispersal, and territory acquisition in the Seychelles warbler. Behav. Ecol. 18, 874–879 (2007).

[b21] Griesser M., Nystrand M. & Ekman J. Reduced mortality selects for family cohesion in a social species. Proc. R. Soc. Lond. B Biol. Sci. 273, 1881–1886 (2006). 10.1098/rspb.2006.3527 PMC1634772 16822747

[b22] Gillis E. A. & Krebs C. J. Survival of Dispersing versus Philopatric Juvenile Snowshoe Hares: Do Dispersers Die? Oikos 90, 343–346 (2000).

[b23] Waser P. M., Nichols K. M. & Hadfield J. D. Fitness consequences of dispersal: Is leaving home the best of a bad lot? Ecology 94, 1287–1295 (2013). 23923492 10.1890/12-1037.1

[b24] Jones W. T. Density-Related Changes in Survival of Philopatric and Dispersing Kangaroo Rats. Ecology 69, 1474–1478 (1988).

[b25] Drickamer L. C. & Vessey S. H. Group changing in free-ranging male rhesus monkeys. Primates 14, 359–368 (1973).

[b26] Colvin J. D. In The Cayo Santiago Macaques. History, behavior and biology (eds Rawlins R. G. & Kessler M. J.) 131–157 (State University of New York Press, 1986).

[b27] Lindburg D. G. Rhesus monkeys: mating season mobility of adult males. Science 166, 1176–1178 (1969). 17775579 10.1126/science.166.3909.1176

[b28] Dubuc C., Ruiz-Lambides A. & Widdig A. Variance in male lifetime reproductive success and estimation of the degree of polygyny in a primate. Behav. Ecol. 25, 878–889 (2014). 25024637 10.1093/beheco/aru052 PMC4095946

[b29] Berard J. D. Life histories of male Cayo Santiago macaques. P. R. Health Sci. J. 8, 61–64 (1989). 2780969

[b30] Meikle D. B. & Vessey S. H. Nepotism among rhesus monkey brothers. Nature 294, 160–161 (1981). 7197754 10.1038/294160a0

[b31] Kulik L., Langos D. & Widdig A. Mothers make a difference: Mothers develop weaker bonds with immature sons than daughters. PLOS ONE 10.1371/journal.pone.0154845 (2016). PMC4871456 27191403

[b32] Trefilov A., Berard J. D., Krawczak M. & Schmidtke J. Natal dispersal in rhesus macaques is related to serotonin transporter gene promoter variation. Behav. Genet. 30, 295–301 (2000). 11206084 10.1023/a:1026597300525

[b33] Krawczak M. . Male Reproductive Timing in Rhesus Macaques Is Influenced by the 5HTTLPR Promoter Polymorphism of the Serotonin Transporter Gene. Biol. Reprod. 72, 1109–1113 (2005). 15635127 10.1095/biolreprod.104.038059

[b34] Alberts S. C. & Altmann J. Balancing costs and opportunities: dispersal in male baboons. Am. Nat. 145, 279–306 (1995).

[b35] Grafen A. In Reproductive Success: Studies of Individual Variation in Contrasting Breeding Systems (ed. Clutton-Brock T.) 454–471 (University Of Chicago Press, 1988).

[b36] Datta S. The acquisition of dominance among free-ranging rhesus monkey siblings. Anim. Behav. 36, 754–772 (1988).

[b37] Bercovitch F. B. Dominance rank and reproductive maturation in male rhesus macaques (*Macaca mulatta*). J. Reprod. Fertil. 99, 113–120 (1993). 8283427 10.1530/jrf.0.0990113

[b38] Gerloff U., Hartung B., Fruth B., Hohmann G. & Tautz D. Intracommunity relationships, dispersal pattern and paternity success in a wild living community of Bonobos (*Pan paniscus*) determined from DNA analysis of faecal samples. Proc. R. Soc. B Biol. Sci. 266, 1189–1195 (1999). 10.1098/rspb.1999.0762 PMC1689947 10406131

[b39] Surbeck M., Mundry R. & Hohmann G. Mothers matter! Maternal support, dominance status and mating success in male bonobos (*Pan paniscus*). Proc. R. Soc. Lond. B Biol. Sci. 278, 590–598 (2011). 10.1098/rspb.2010.1572 PMC3025686 20810444

[b40] VanderWaal K. L., Mosser A. & Packer C. Optimal group size, dispersal decisions and postdispersal relationships in female African lions. Anim. Behav. 77, 949–954 (2009).

[b41] Travis J. M. J., Murrell D. J. & Dytham C. The evolution of density–dependent dispersal. Proc. R. Soc. Lond. B Biol. Sci. 266, 1837–1842 (1999).

[b42] van Noordwijk M. A. & van Schaik C. P. In Sexual selection in primates (eds Kappeler P. M. & van Schaik C. P.) 208–229 (Cambridge University Press, 2004).

[b43] Soltis J. . Sexual selection in Japanese macaques II: Female mate choice and male-male competition. Anim. Behav. 54, 737–746 (1997). 9299057 10.1006/anbe.1997.0568

[b44] Debeffe L., Richard E., Medill S. A., Weisgerber J. N. & McLoughlin P. D. Costs of social dispersal in a polygynous mammal. Behav. Ecol. 26, 1476–1485 (2015).

[b45] Berard J. D., Nurnberg P., Epplen J. T. & Schmidtke J. Alternative Reproductive Tactics and Reproductive Success in Male Rhesus Macaques. Behaviour 129, 177–201 (1994).

[b46] Berard J. A four-year study of the association between male dominance rank, residency status, and reproductive activity in rhesus macaques (*Macaca mulatta*). Primates 40, 159–175 (1999). 23179538 10.1007/BF02557708

[b47] Manson J. H. Evolved psychology in a novel environment. Hum. Nat. 9, 97–117 (1998). 26197442 10.1007/s12110-998-1000-7

[b48] Bercovitch F. B. Reproductive strategies of rhesus macaques. Primates 38, 247–263 (1997).

[b49] Soltis J., Thomsen R. & Takenaka O. The interaction of male and female reproductive strategies and paternity in wild Japanese macaques. Macaca fuscata. Anim. Behav. 62, 485–494 (2001).

[b50] Schülke O., Bhagavatula J., Vigilant L. & Ostner J. Social bonds enhance reproductive success in male macaques. Curr. Biol. 20, 2207–2210 (2010). 21093261 10.1016/j.cub.2010.10.058

[b51] Bauers K. A. & Hearn J. P. Patterns of paternity in relation to male social rank in the stumptailed macaque, Macaca arctoides. Behaviour 129, 149–176 (1994).

[b52] Blyton M. D. J., Banks S. C. & Peakall R. The effect of sex-biased dispersal on opposite-sexed spatial genetic structure and inbreeding risk. Mol. Ecol. 24, 1681–1695 (2015). 25761248 10.1111/mec.13149

[b53] Isvaran K. & Clutton-Brock T. Ecological correlates of extra-group paternity in mammals. Proc. R. Soc. B-Biol. Sci. 274, 219–224 (2007). 10.1098/rspb.2006.3723 PMC1685851 17148250

[b54] Berard J. D., Nürnberg P., Epplen J. T. & Schmidtke J. Male rank, reproductive behavior, and reproductive success in free-ranging rhesus macaques. Primates 34, 481–489 (1993).

[b55] Langos D., Kulik L., Mundry R. & Widdig A. The impact of paternity on male–infant association in a primate with low paternity certainty. Mol. Ecol. 22, 3638–3651 (2013). 23682587 10.1111/mec.12328 PMC3800749

[b56] Jungwirth A., Walker J. & Taborsky M. Prospecting precedes dispersal and increases survival chances in cooperatively breeding cichlids. Anim. Behav. 106, 107–114 (2015).

[b57] *The Cayo Santiago macaques*. History, behavior and biology (State University of New York Press, 1986).

[b58] Marriott B. M., Roemer J. & Sultana C. An overview of the food intake patterns of the Cayo Santiago rhesus monkeys (*Macaca mulatta*): report of a pilot study. P. R. Health Sci. J. 8, 87–94 (1989). 2780973

[b59] Hernández-Pacheco R. . Demographic variability and density-dependent dynamics of a free-ranging rhesus macaque population. Am. J. Primatol. 75, 1152–1164 (2013). 23847126 10.1002/ajp.22177 PMC3920185

[b60] Strier K. B. Primate behavioral ecology (Pearson Education, Inc., 2006).

[b61] Kulik L., Amici F., Langos D. & Widdig A. Sex differences in the development of aggressive behavior in rhesus macaques (*Macaca mulatta*). Int. J. Primatol. 36, 764–789 (2015). 10.1007/s10764-015-9826-4 PMC4430863 25983360

[b62] Kulik L., Amici F., Langos D. & Widdig A. Sex differences in the development of social relationships in rhesus macaques (*Macaca mulatta*). Int. J. Primatol. 36, 353–376 (2015). 25983360 10.1007/s10764-015-9826-4 PMC4430863

[b63] Widdig A. . Genetic studies on the Cayo Santiago rhesus macaques: A review of 40 years of research. Am. J. Primatol. 78, 44–62 (2016). 26031601 10.1002/ajp.22424

[b64] R Development Core Team. R: A language and environment for statistical computing (R Foundation for Statistical Computing, 2013).

[b65] Barr D. J., Levy R., Scheepers C. & Tily H. J. Random effects structure for confirmatory hypothesis testing: Keep it maximal. J. Mem. Lang. 68, 255–278 (2013). 10.1016/j.jml.2012.11.001 PMC3881361 24403724

