# Supplementary material for: Individual dispersal decisions affect fitness via maternal rank effects in male rhesus macaques
Source: Sci Rep. 2016 Aug 31;6:32212. doi: 10.1038/srep32212 (PMC5006056; doi:10.1038/srep32212)
Supplement: Supplementary Information [file srep32212-s1.doc]

**Supplementary material for *"Individual dispersal decisions affect fitness via maternal rank effects in male rhesus macaques"* by Weiß, Kulik, Ruiz-Lambides & Widdig**

Supplementary Table S1: Results of the GLMMs investigating the relationship between natal dispersal age and five fitness traits. Survival (N = 840) and post-dispersal longevity (N = 402) were fitted as binomial responses, probability to reproduce (N = 297), age at first reproduction (N = 252) and LRS (N = 84) as were fitted with a Poisson error structure. Predictors with p < 0.05 are marked in bold.

| trait | Predictor | Estimate | SE | z | p |
| --- | --- | --- | --- | --- | --- |
| *survival (1st year after dispersal)* | Intercept | 7.9502 | 2.142 |  |  |
|  | natal dispersal age | 0.0001 | 0.001 | 0.128 | 0.898 |
|  | maternal rank | -0.2439 | 1.805 | -0.135 | 0.893 |
|  | co-residence with mother | -0.0007 | 0.001 | -0.463 | 0.643 |
| *post-dispersal longevity (years)* | Intercept | 1.6914 | 0.489 |  |  |
|  | natal dispersal age | -0.0001 | 0.0003 | -0.469 | 0.639 |
| *probability to reproduce* | Intercept | -0.6615 | 1.363 |  |  |
|  | **natal dispersal age** | **0.001** | **0.0004** | **2.424** | **0.015** |
|  | maternal rank | 0.5904 | 0.605 | 0.976 | 0.329 |
|  | co-residence with mother | 0.0004 | 0.001 | 0.309 | 0.757 |
| *age at 1st reproduction (years)* | Intercept | 2.0280 | 0.198 |  |  |
|  | natal dispersal age | -0.00003 | 0.000 | -0.750 | 0.454 |
|  | maternal rank | -0.1104 | 0.089 | -1.247 | 0.212 |
|  | co-residence with mother | 0.00001 | 0.000 | 0.074 | 0.941 |
| *LRS* | Intercept | 0.6308 | 0.276 |  |  |
|  | **natal dispersal age** | **0.0005** | **0.0001** | **3.651** | **< 0.001** |
|  | **maternal rank** | **0.7064** | **0.292** | **2.420** | **0.016** |

Random intercepts were fitted for maternal ID, birth group and cohort. For survival, probability to reproduce and age at first reproduction and LRS, random slopes were fitted for natal dispersal age and maternal rank within birth group and cohort, for post-dispersal longevity random slopes were fitted for natal dispersal age within birth group and cohort.

Supplementary methods

We fitted Linear Mixed Models (LMM) and Generalized Linear Mixed Models (GLMM) using the R-package "lme4" (version 1.0-6^1^).

*Variation in dispersal age*

To identify variables related to variation in dispersal age we log-transformed natal dispersal to fit model assumptions of a Gaussian response. As measures of the population environment we fitted population size and the number of groups on CS when the focal reached dispersal age (i.e. three years of age). As measures of the group environment we fitted adult group size and adult sex ratio when the focal was three years old. As measures of the maternal environment we used maternal rank, maternal family size (i.e. number of adult female relatives up to 1^st^ cousins at focal age 3) and, as a proxy for maternal experience, the number of offspring (surviving the first year) produced by the mother until a focal male's birth. We further scored if the focal had familiar older brothers that had already dispersed when the focal reached dispersal age and accounted for orphaned focals by counting the number of days the mother was present in the first 1000 days of life. As we expected the effects of some predictors to be dependent on other predictors we fitted interactions between adult group size and adult sex ratio, adult group size and maternal family size and between population size and the number of groups on CS.

To control for the possibility that males born early in the season would also disperse earlier than those born late in the season we fitted the age at the onset of the mating season in the focal's third year of life as a fixed effects control predictor. We chose this measure rather than the male's birth date relative to the onset of the birth season to account for the steady progressing of the mating and birth season on CS over the years ^2^. We further fitted the identity of the mother, the male's birth group and cohort as random effects. To avoid underestimating p-values of within subjects test predictors we fitted random slopes for all predictors that varied within each level of a random effect (see Tab. 1; ^3^). For this purpose we excluded the nine males born into group O from the analysis because some predictors did not vary within this random effects level. This reduced the total number of males used in the model to 912. All covariates were z-transformed to a mean of zero and a standard deviation of one to get comparable estimates and facilitate model convergence ^4^.

We fitted the models using Maximum Likelihood (rather than Restricted Maximum Likelihood; ^5^) to achieve more reliable p-values. Model assumptions of normally distributed and homogeneous residuals were checked visually, which did not indicate any obvious deviation from these assumptions. To check for collinearity we determined Variance Inflation Factors (VIF, ^6^) for a standard linear model excluding the random effects using the function "vif" in the package "car" ^7^. VIFs were below 1.48 in all cases and thus indicated collinearity to be no issue. We assessed model stability by excluding subjects from the data one at a time. The estimates of these sets of models were similar to the coefficients of the original model, indicating that no influential cases exist. We assessed the significance of the full model by comparing it to a null model comprising only the control predictor, random intercepts and random slopes using a Likelihood Ratio Test (R function "anova", test = "Chisq"). P-values for the individual effects were obtained with Likelihood Ratio Tests using the function "drop1". We removed interactions with a p-value < 0.1 from the model to facilitate the interpretation of main terms but kept all other terms in the model.

*Natal dispersal age and fitness*

For investigating survival to one year after dispersal we used all focal males that remained on CS for a full year after dispersal or died a natural death within the year (n = 840). For post-dispersal longevity we used all focal males that had died naturally on CS before 2014 (n = 402). As paternity was only systematically assessed for offspring from 1992 onwards, we restricted analyses of reproductive success to males maturing after 1992 (i.e. from cohorts 1989 or later). The probability to reproduce was assessed for males who had sired offspring or had died naturally on CS without siring offspring (n = 297). Data on age at first reproduction were available for 254 focal males. LRS was assessed for 87 males that reproduced and died naturally on CS before 2014.

For each fitness trait we calculated a GLMM using the function "glmer". Survival of the first year after dispersal and the probability to reproduce were fitted as binomial response variables (yes/no), post-dispersal longevity (in years), age at first reproduction (in years) and LRS were fitted with a Poisson error structure. Age at natal dispersal (in days) was fitted as the only test predictor. As control predictors we included individual traits that tended to affect age at natal dispersal, i.e. maternal rank and co-residence with the mother in order to assess whether fitness traits were affected by age at natal dispersal or a phenotypically correlated trait. In all models we further included identity of the mother, the male's birth group and cohort as random effects and fitted all possible random slopes. For this purpose we excluded the only males from cohort 2008 and group Q from the analysis of age at first reproduction (resulting in an n of 252) to allow fitting random slopes within the respective random effects. For the same reason we removed the only males from cohorts 1999 and 2006 as well as from group Q from the analysis of LRS, reducing the n for this analysis to 84. None of the models were overdispersed (dispersion parameters 0.18 – 1.07) and variance inflation factors indicated collinearity to be no issue (all VIFs < 1.12). We assessed model stability by excluding random effects levels from the data one at a time. The model on post-dispersal longevity was highly instable when the control predictors were included in the model; we therefore ran this model without control predictors but including all random terms. Models on the other fitness traits and the model on post-dispersal longevity without control predictors produced stable results when single levels were excluded. We assessed the significance of natal dispersal age using the z and corresponding P value provided by the function "glmer".

*Natal dispersal age, place of reproduction and group choice*

To investigate the relationship between natal dispersal age and where males first reproduced we used focal males born in cohort 1989 or later with known age of first reproduction. We excluded the only male born in group Q and the only male from cohort 2008 from the analysis to allow the inclusion of natal dispersal age within birth group and cohort as a random slope (resulting in an N of 252 males). The GLMM was slightly underdispersed (dispersion parameter 0.53) and removal of subjects one at a time indicated no influential cases to exist. We assessed the significance of natal dispersal age using the z and corresponding P value provided by the function "glmer".

Detailed information on reproduction before natal dispersal was available for 94 males, for whom we determined if they had sired offspring with females from their natal group (i.e. within-group offspring) or from other groups (i.e. extra-group offspring). We considered infants to be extra-group offspring if the male resided in another group than the infant's mother on the day the infant was conceived. The conception date was determined by subtracting the mean gestation length of 166.5 ^8^ from the infant's birth date. Because gestation length may vary by some days (SD of 7.4 days ^8^) we did not consider infants with an assumed conception date of ± 10 days of the natal dispersal date.

The model testing if having fathered offspring in another group affected group choice during natal dispersal was slightly underdispersed (dispersion parameter 0.713) and the model was stable with regards to the removal of single cases. As in the other GLMMs, the significance of the test predictor was assessed using the z and corresponding P value provided by the function "glmer".

1. Bates, D., Maechler, M., Bolker, B. & Walker, S. *lme4: Linear mixed-effects models using Eigen and S4*. (2014).

2. Hernández-Pacheco, R. *et al.* Discovery of a secular trend in Cayo Santiago macaque reproduction. *Am. J. Primatol.* **78,** 227–237 (2016).

3. Barr, D. J., Levy, R., Scheepers, C. & Tily, H. J. Random effects structure for confirmatory hypothesis testing: Keep it maximal. *J. Mem. Lang.* **68,** 255–278 (2013).

4. Schielzeth, H. Simple means to improve the interpretability of regression coefficients. *Methods Ecol. Evol.* **1,** 103–113 (2010).

5. Bolker, B. M. *et al.* Generalized linear mixed models: a practical guide for ecology and evolution. *Trends Ecol. Evol.* **24,** 127–135 (2009).

6. Field, A. *Discovering statistics using SPSS*. (Sage Publications, London, 2005).

7. Fox, J. & Weisberg, S. *An {R} Companion to Applied Regression, Second Edition*. (Sage, 2011).

8. Silk, J. B., Short, J., Roberts, J. & Kusnitz, J. Gestation length in rhesus macaques (*Macaca mulatta*). *Int. J. Primatol.* **14,** 95–104 (1993).
